# Supplementary material for: Treatment of Diabetic Neuropathy with A Novel PAR1-Targeting Molecule
Source: Biomolecules. 2020 Nov 13;10(11):1552. doi: 10.3390/biom10111552 (PMC7698286; doi:10.3390/biom10111552)
Supplement: Supplementary file 1 [file biomolecules-10-01552-s001.pdf]

Supplementary materials

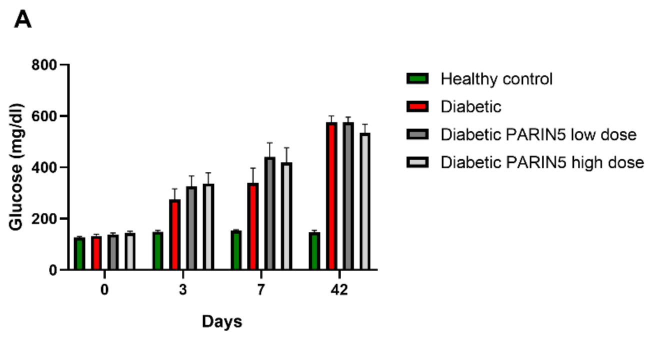

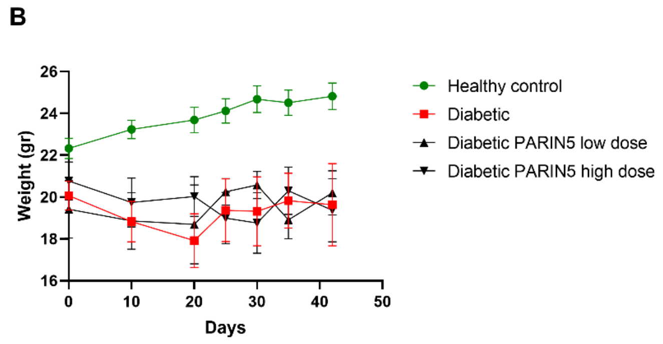

Figure S1. Glucose levels and weight in diabetic mice: (A) STZ injected mice developed glucose levels above 250 mg/dL. PARIN5 treatment did not influence blood glucose levels. (B) diabetic mice showed significantly decreased body weights, starting at day 10. PARIN5 did not have a significant effect on weight.
